# Adsorptive removal of lead, copper, and nickel using natural and activated Egyptian calcium bentonite clay

**DOI:** 10.1038/s41598-025-95184-7

**Published:** 2025-04-16

**Authors:** Mahmoud I. Eleraky, Taha M. A. Razek, Ibrahim W. Hasani, Yosri A. Fahim

**Affiliations:** 1 Central Laboratories of the Egyptian Mineral Resources Authority, Cairo, Egypt; 2 Faculty of Graduate Studies and Environmental Research, Ain Shams University, Cairo, Egypt; 3 Department of Pharmaceutics, Faculty of Pharmacy, S.P.U., M.P.U and Idlib University, Idlib, Syria; 4 Health Sector, Faculty of Science, Galala University, Galala City, Suez, 43511 Egypt

**Keywords:** Bentonite clay, Adsorption capacity, Adsorption, Alkali activation, Environmental sciences, Chemistry

## Abstract

This study evaluates the efficiency of alkali-activated Egyptian calcium bentonite, obtained from the El Alamein region in northern Egypt, for the removal of copper (Cu^2⁺^), lead (Pb^2⁺^), and nickel (Ni^2⁺^) from synthetic wastewater. The bentonite samples underwent a series of preparation steps, including crushing, ball milling, magnetic separation, acid treatment with 0.1N acetic acid, and alkali activation using 5% sodium carbonate (Na_2_CO_3_). Various analytical techniques, such as X-ray fluorescence (XRF), X-ray diffraction (XRD), Fourier-transform infrared spectroscopy (FTIR), cation exchange capacity (CEC) measurements, scanning electron microscopy (SEM), and free swelling analysis, were employed to characterize the materials. Absorption experiments were performed to examine the effects of pH, temperature, starting metal concentration, bentonite dose, and contact duration on heavy metal removal. The characterization results confirmed that montmorillonite was the predominant mineral in both the natural and activated bentonite samples. Adsorption studies indicated a significant improvement in heavy metal removal efficiency after activation. Under optimal conditions (pH 7, 1 g/L adsorbent dose, 120 min contact time, 20 mg/L initial metal concentration, and 20 °C), the maximum adsorption capacities of the activated bentonite were determined as 14 ± 0.03 mg/g for Cu^2+^, 13 ± 0.04 mg/g for Pb^2+^, and 12.2 ± 0.05 mg/g for Ni^2+^, exceeding those of the natural bentonite, which recorded capacities of 9.2 ± 0.04 mg/g, 9 ± 0.03 mg/g, and 8 ± 0.02 mg/g, respectively. Adsorption equilibrium data according to the Langmuir isotherm model, exhibiting high correlation values (R^2^ = 0.9979 for Cu^2+^, 0.9972 for Pb^2+^, and 0.9973 for Ni^2+^). Moreover, kinetic modeling demonstrated that the adsorption followed a pseudo-second-order mechanism, suggesting an intense chemisorption process. The thermodynamic analysis indicated that the adsorption process was spontaneous and endothermic, demonstrating enhanced adsorption at higher temperatures.

## Introduction

The increasing release of heavy metals into the environment due to industrial expansion and human activities has become a significant issue, leading to contamination of water, air, and soil. These pollutants pose a serious threat to living organisms as they accumulate in biological systems over time^1^. In plants, heavy metals disrupt essential physiological processes such as nutrient uptake and photosynthesis, negatively affecting growth and yield. In humans and animals, chronic exposure to these elements has been linked to various health disorders, including organ damage, kidney dysfunction, and carcinogenesis^2^. One of the primary pathways for heavy metal contamination in aquatic environments is through industrial discharge, particularly from sectors such as electroplating, mining, battery production, and fertilizer manufacturing^3^. Without proper treatment, these pollutants persist in ecosystems, causing long-term environmental damage. Therefore, efficient treatment methods are essential to remove heavy metals from wastewater before its release into natural water bodies^4^. Several techniques have been developed for the removal of heavy metals from industrial effluents, including chemical precipitation, ion exchange, membrane filtration, and adsorption^5^. Adsorption is recognized as one of the most effective and economical approaches due to its simplicity and high removal efficiency^6^. Bentonite clay, known for its natural ability to adsorb contaminants, has been extensively investigated for wastewater treatment applications^7^.

Bentonite, a clay mineral dominated by montmorillonite, is extensively used in diverse fields, including agriculture, petroleum refining, and environmental remediation. Its high cation exchange capacity (CEC) and strong adsorption affinity for pollutants make it a promising material for water treatment applications. However, raw bentonite often contains various impurities, such as quartz, kaolinite, and feldspar, which can interfere with its adsorption efficiency^8^. Additionally, the type of exchangeable cations present in the bentonite structure influences its swelling properties and adsorption behavior. Among different bentonite types, sodium montmorillonite exhibits superior performance due to its higher swelling ability and enhanced ion exchange capacity, making it particularly effective for pollutant removal^9^.

Alkali activation using sodium carbonate (Na_2_CO_3_) is a widely used technique to improve the adsorption characteristics of bentonite by converting calcium-rich bentonite into sodium-rich bentonite^10^. This chemical modification enhances the material’s cation exchange capacity, improves its swelling properties, and facilitates better dispersion in aqueous systems, thereby increasing its efficiency in metal ion adsorption. Furthermore, alkali activation introduces additional active sites on the clay surface, which further strengthens its ability to remove heavy metal contaminants from water^11^. These structural and compositional modifications make alkali-activated bentonite a highly effective and sustainable adsorbent for wastewater treatment applications. Compared to other bentonite modification methods, alkali activation is a simple, low-cost approach that requires minimal energy input while significantly improving adsorption performance. Alternative processes, such as the extrusion sodium treatment, enhance the swelling and rheological properties of bentonite but require extensive mechanical processing and higher energy consumption^12^. While the extrusion technique is beneficial for industrial applications like drilling fluids, it is less practical for adsorption-based water treatment. In contrast, alkali activation is a more efficient and accessible method, making it suitable for large-scale industrial wastewater treatment as well as decentralized water purification systems^13^.

This study aims to investigate the efficiency of sodium carbonate-modified Egyptian calcium bentonite for heavy metal removal, addressing the urgent need for cost-effective and sustainable remediation technologies. Utilizing naturally abundant and locally available bentonite provides an environmentally friendly alternative to expensive synthetic adsorbents, contributing to sustainable water treatment solutions.

## Materials and methods

### Materials

The bentonite clay sample used in this research was collected from Egypt’s northern coastal region. Sodium carbonate (Na_2_CO_3_) and Acetic Acid (CH_3_COOH) supplied by ADVENT Chem Bio was employed for the clay activation and treatment process. The heavy metal sources utilized in the study included lead nitrate (Pb(NO_3_)_2_), copper nitrate trihydrate (Cu(NO_3_)_2_ ·3H_2_O), and nickel nitrate hexahydrate (Ni(NO_3_)_2_ ·6H_2_O), all obtained from ADVENT Chem Bio. All reagents were of analytical grade and were used without further purification. Throughout the experimental procedures, Ultrapure Milli-Q water served as the solvent.

### Activation of bentonite

**Fig. 1:**
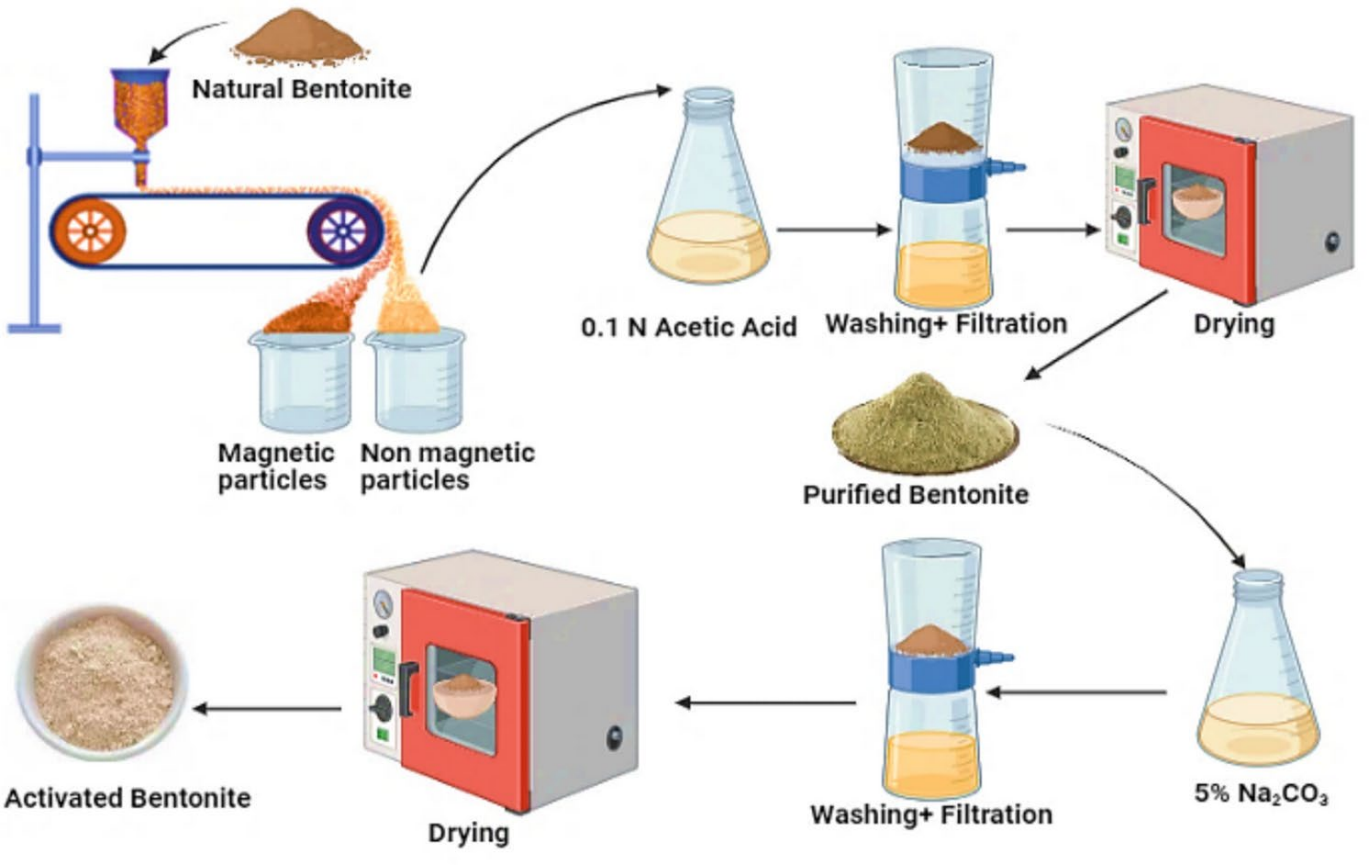
Activation process of natural bentonite.

The raw Egyptian bentonite was initially crushed and ground using a ball mill, followed by sieving through a 63 μm mesh to obtain a uniform particle size. To minimize iron impurities, magnetic separation was performed. The material then underwent a purification step using 0.1 N acetic acid at ambient temperature to eliminate carbonate content. After purification, the sample was thoroughly washed with water and dried at 90 °C. For alkali activation, the purified bentonite was treated with a 5% sodium carbonate (Na_2_CO_3_) solution. A 20 g sample was placed in a conical flask, and 100 mL of 5% Na_2_CO_3_ solution was added, maintaining a solid-to-liquid ratio of 1:5. The mixture was subsequently filtered, rinsed with distilled water until a neutral pH of 7 was achieved, and dried again at 90 °C^14^, as shown in Fig. 1.

### Characterization of natural and activated bentonite samples

#### X‑ray fluorescence (XRF)

The chemical composition of natural and activated bentonite samples was assessed using X-ray fluorescence (XRF, ZSX Primus IV, Rigaku), which determined the percentage of oxides in the samples.

#### X‑ray diffraction (XRD)

The crystalline phases of the samples were analyzed at ambient temperature using a Phillips X’Pert PRO X-ray diffractometer. This instrument operated with a Cu Kα radiation source at 40 kV and 40 mA. A graphite monochromator was applied to the secondary beam. Data collection was conducted over a 2θ range spanning 10°–80°, with a step size of 0.02° and a counting time of 2 s per step.

#### Fourier‑transform infrared spectroscopy (FTIR)

Fourier-Transform Infrared (FTIR) spectroscopy was employed to examine the functionalized surface of both natural and activated bentonite samples. The FTIR analysis was conducted using the Cary 630 spectrometer from Agilent Technologies, with the spectrum range analyzed between 4000 and 400 cm^−1^.

#### Swelling index

The swelling index was assessed following the ASTM D5 standard. A 2 g portion of dried, finely milled bentonite was introduced into a 100 mL graduated cylinder in small increments of 0.1 g. Each portion was given at least 10 min to fully hydrate and settle before the next addition. This process was repeated until the entire 2 g sample was incorporated. The cylinder was then sealed and left undisturbed for a period ranging from 16 to 24 h to allow the swelling process to reach equilibrium. The final volume was recorded once stabilization was achieved^15^.

#### Cation exchange capacity (CEC)

The cation exchange capacity (CEC) was evaluated using barium chloride (BaCl_2_) as the saturating agent. This procedure involved saturating the clay’s exchange sites with barium ions until equilibrium was achieved, followed by ethanol washing to remove any excess barium, and subsequently replacing it with ammonium. Initially, the bentonite samples were thoroughly rinsed with deionized water. Subsequently, 1 g of the desiccated sample was dispersed in 10 mL of 0.5 N BaCl_2_·2H_2_O solution and agitated on a reciprocating shaker for 30 min. The suspension was then vacuum-filtered using Whatman No. 5 filter paper. To ensure complete saturation, the sample was leached with 100 mL of 1 N BaCl_2_·2H_2_O, followed by ethanol washing (200 mL) to eliminate any residual barium chloride. The exchangeable barium was then displaced by rinsing the sample in a clean flask with 225 mL of 1 N ammonium acetate solution (pH 7). The resulting leachate was diluted to a total volume of 250 mL using deionized water in a volumetric flask. The final barium ion concentration was determined through Inductively Coupled Plasma Optical Emission Spectroscopy (ICP-OES)^16^.

#### Scanning electron microscope (SEM)

The natural and activated bentonite surface properties were examined using a Quanta 250 FEG scanning electron microscope (SEM) integrated with an energy-dispersive X-ray Spectroscopy (EDX) system. The microscope functioned at an accelerating voltage of 30 kV, capturing images at magnifications ranging from 14 × to 1,000,000 × . This technique provided detailed insights into the structural differences between the samples before and after activation.

#### Point of zero charge (Pzc)

To determine the point of zero charge (pzc) for both natural and activated bentonite, 50 mL of a 0.1 M NaCl solution was placed in a sealed Erlenmeyer flask. The pH of the solution was adjusted within the range of 2–12 by carefully adding 0.01 M HCl or NaOH. The initial pH value was recorded using a THERMO SCIENTIFIC ORION STAR A111 pH meter^17^. Next, 0.1 g of the bentonite sample was introduced into the solution, and the mixture was continuously stirred at 300 rpm for 24 h to achieve equilibrium. After the designated time, the final pH was measured and documented. A graph plotting the final pH against the initial pH was generated, and the pzc was identified as the point where the difference between the final and initial pH (ΔpH) equaled zero.

### General adsorption procedure of synthetic wastewater

**Fig. 2:**
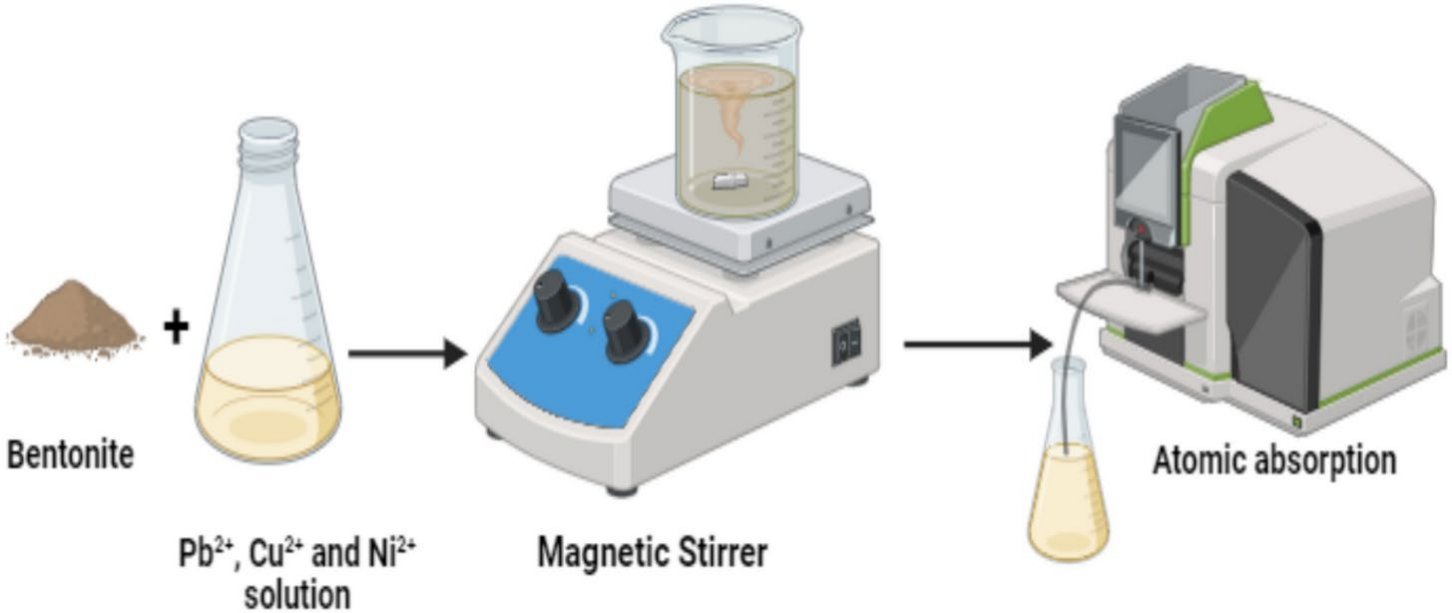
Adsorption process of heavy metals by bentonite sample.

Aqueous solutions of 1000 mg/L Cu^2⁺^, Pb^2⁺^, and Ni^2⁺^ were prepared by dissolving exact amounts of Cu(NO_3_)_2_ ·3H_2_O, Pb(NO_3_)_2_, and Ni(NO_3_)_2_ ·6H_2_O in 1000 mL of deionized water to achieve the desired concentrations of each metal ion. The adsorption of Cu^2^⁺, Pb^2^⁺, and Ni^2+^ by natural and activated bentonite samples was investigated under various conditions, including pH, bentonite dosage, initial concentration, temperature, and contact time. The concentrations of metal ions in the solutions were evaluated using the GBC SavantAA Atomic Absorption Spectrometer, as illustrated in Fig. 2. The amount of adsorbed heavy metals per unit mass of activated bentonite (qe) was calculated using the following equations^18^:

1 $${\text{q}}_{{\text{e}}} = \left( {{\text{C}}_{{\text{o}}} {-}{\text{C}}_{{\text{e}}} } \right).\frac{V}{m}$$

where C_o_ (mg/L) represents the initial concentration of heavy metal ions, C_e_ (mg/L) is the equilibrium concentration of heavy metal ions, V (L) is the volume of the aqueous solution, and m (g) is the mass of bentonite. All experiments were performed in duplicate, and the average values were used for subsequent calculations.

### Thermodynamic studies

The thermodynamic characteristics associated with cation adsorption on activated bentonite, including Gibbs free energy (ΔG), enthalpy (ΔH), and entropy (ΔS), were computed using the relevant mathematical formulas^19^:

2 $$\Delta {\text{G}}^\circ = - {\text{RT ln K}}_{{\text{L}}}$$

where K_L_ represents the equilibrium constant derived from the Langmuir model, T is the absolute temperature (K), and R denotes the universal gas constant (8.314 × 10^−3^ kJ K^−1^ mol^−1^). The relationship between the equilibrium constant and the thermodynamic parameters of enthalpy and entropy is expressed using the Van’t Hoff equation^20^:

3 $$\text{ln KL }=\frac{{\Delta {\text{S}}}^\circ }{\text{R}}-\frac{{\Delta {\text{H}}}^\circ }{\text{RT}}$$

To evaluate the thermodynamic behavior of the adsorption process, experiments were conducted at five different temperatures: 293 K, 303 K, 313 K, 323 K, and 333 K. The pH of the solution was maintained at 7.0 for the adsorption of Cu^2+^, Pb^2⁺^, and Ni^2⁺^ ions. Each experiment was performed using 1 g/L of bentonite in a 50 mL solution containing the respective metal ions.

### Statistical analysis

The statistical analysis of the experimental data was conducted using SPSS software version 15. All adsorption experiments including pH, adsorbent dosage, temperature, and initial metal concentration were performed in triplicate, and the results were expressed as mean ± standard deviation (SD). The standard deviation was calculated to assess the variability and reliability of the data.

## Results and discussion

### Characterization of natural and activated bentonite samples

#### X‑ray fluorescence (XRF)

The findings revealed an improvement in the properties of the activated bentonite (AB). As shown in Table 1, the iron content was reduced as a result of the magnetic separation process. The chemical analysis indicated that calcium montmorillonite was the primary clay mineral present in the natural bentonite sample, as evidenced by its calcium content. However, after activation, calcium levels decreased while sodium concentration increased. Additionally, the reduction in iron content in the activated sample further confirmed the effectiveness of the magnetic separation technique.

**Table 1:** XRF analysis of Natural and Activated bentonite samples.

Composition (%)	Natural bentonite	Activated bentonite
SiO_2_	51.1	49
TiO_2_	1.76	2.05
Al_2_O_3_	17.25	18.7
Fe_2_O_3_	8.97	8.15
MnO_2_	0.21	0.09
MgO	1.9	1.5
CaO	1.55	0.88
Na_2_O	0.7	2.64
K_2_O	1.75	1.58
P_2_O_5_	0.1	0.1
SO_3_	0.25	0.23
L.O.I	14.45	14.88

#### X‑ray diffraction (XRD)

**Fig. 3:**
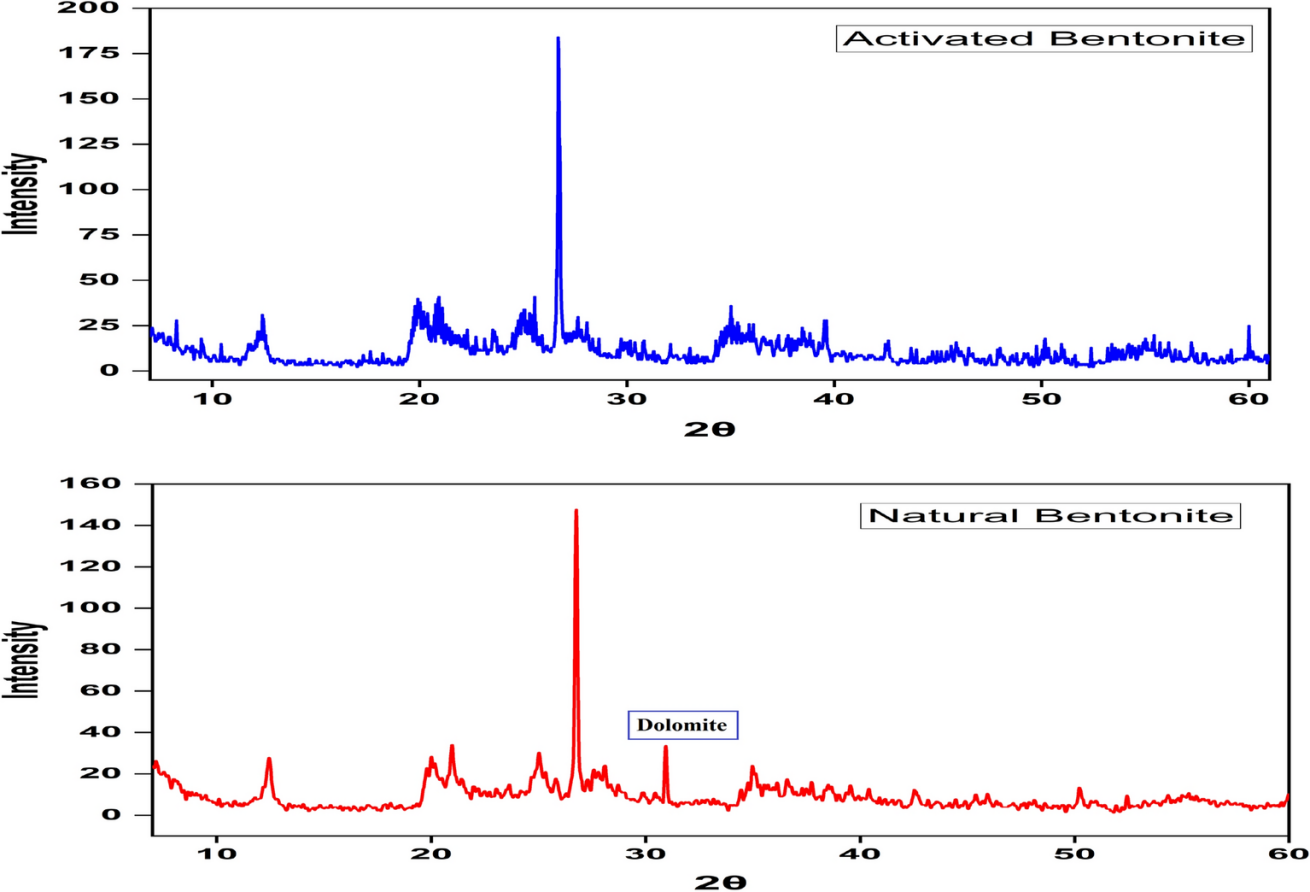
XRD analysis for natural and activated bentonite samples.

XRD analysis of the natural and activated bentonite samples confirmed that montmorillonite was the primary mineral, accompanied by smaller amounts of kaolinite and quartz. The absence of the dolomite peak in the activated sample suggests its removal during the acetic acid treatment, as illustrated in Fig. 3. The structural alterations identified in the XRD patterns of activated bentonite significantly contribute to improving its adsorption capability. The expansion of interlayer spacing following alkali activation, as evidenced by the shift in diffraction peaks, creates more available sites for heavy metal ion uptake. These modifications enhance the material’s surface area and cation exchange capacity (CEC), leading to more effective adsorption of Cu^2⁺^, Pb^2^⁺, and Ni^2^⁺ from aqueous solutions. Additionally, the improved crystallinity and phase composition of activated bentonite strengthen its ability to retain metal ions by supporting monolayer adsorption.

#### Fourier‑transform infrared spectroscopy (FTIR)

**Fig. 4:**
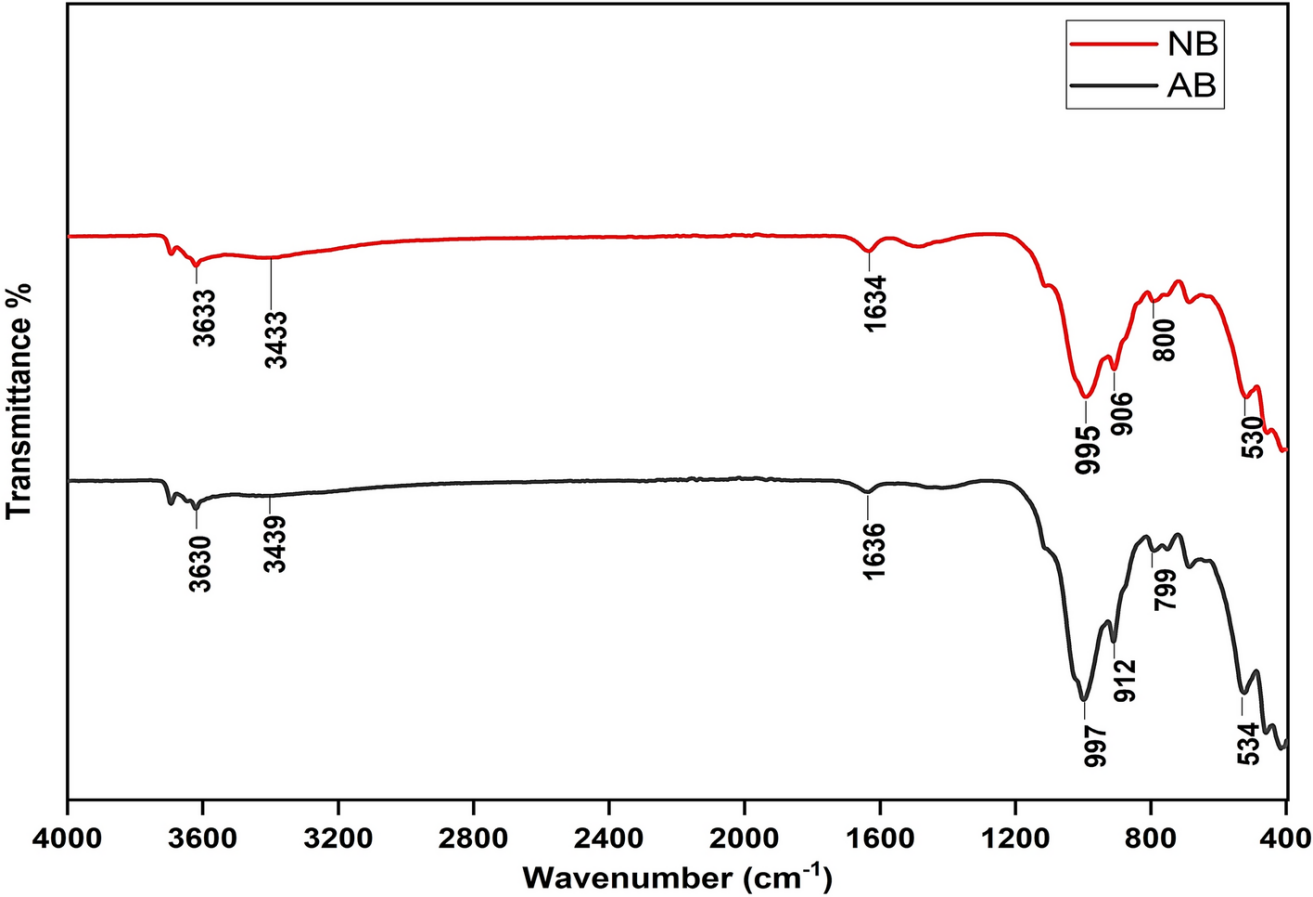
FTIR spectra of natural (NB) and activated (AB) bentonite samples.

The FTIR spectra of natural and activated bentonite samples, presented in Fig. 4, illustrate the presence of characteristic functional groups. Notably, the stretching vibrations associated with Si–O and Si–O–Al bonds appear at 540 cm^-1^^21^, while the peak at 905 cm^−1^ is attributed to Si–O–Si–O–Al stretching^21^. The peak observed at 800 cm^−1^ corresponds to O–Si–O stretching. A broad band near 3433 cm^−1^ is associated with the stretching vibrations of OH or H_2_O molecules, possibly present on the clay surface, and the peak at 1633 cm^−1^ is linked to H–O–H bending vibrations^22^. The O–H stretching vibration of structural hydroxyl groups in montmorillonite appears at 3633 cm^-1^. Furthermore, both natural and activated bentonite samples display distinct peaks near 995 cm^-1^ and 912 cm^-1^, corresponding to Si–O and Al–O stretching vibrations, respectively^23^. The modifications observed in the FTIR spectra after activation indicate the formation of additional hydroxyl (–OH) and silanol (Si–OH) functional groups, which play a vital role in the adsorption mechanism. These functional groups serve as active binding sites for metal ions, promoting both surface complexation and ion exchange. The enhanced presence of hydroxyl groups following activation strengthens the interaction between metal ions and the bentonite surface, ultimately boosting adsorption efficiency.

#### Swelling index

The swelling index results indicate a significant improvement in the bentonite sample after activation. As shown in Table 2, the swelling index of activated bentonite increased to 27 ± 1.1 mL/2 g, compared to 7 ± 0.4 mL/2 g for natural bentonite.

**Table 2:** Cation exchange capacity & swelling index of natural and activated bentonite.

Sample	CEC (Meq/100 g)	Swelling index/2 g (mL/2g)
Natural bentonite	45 ± 1.4	7 ± 0.4
Activated bentonite	60 ± 2.0	27 ± 1.1

#### Cation exchange capacity

The cation exchange capacity (CEC) was evaluated using BaCl_2_ as the saturating cation. As shown in Table 2, activation with 5% Na_2_CO_3_ significantly enhanced the CEC, increasing from 45 ± 1.4 meq/100g in natural bentonite to 60 ± 2.0 meq/100g in activated bentonite.

#### Point of zero charge determination

**Fig. 5:**
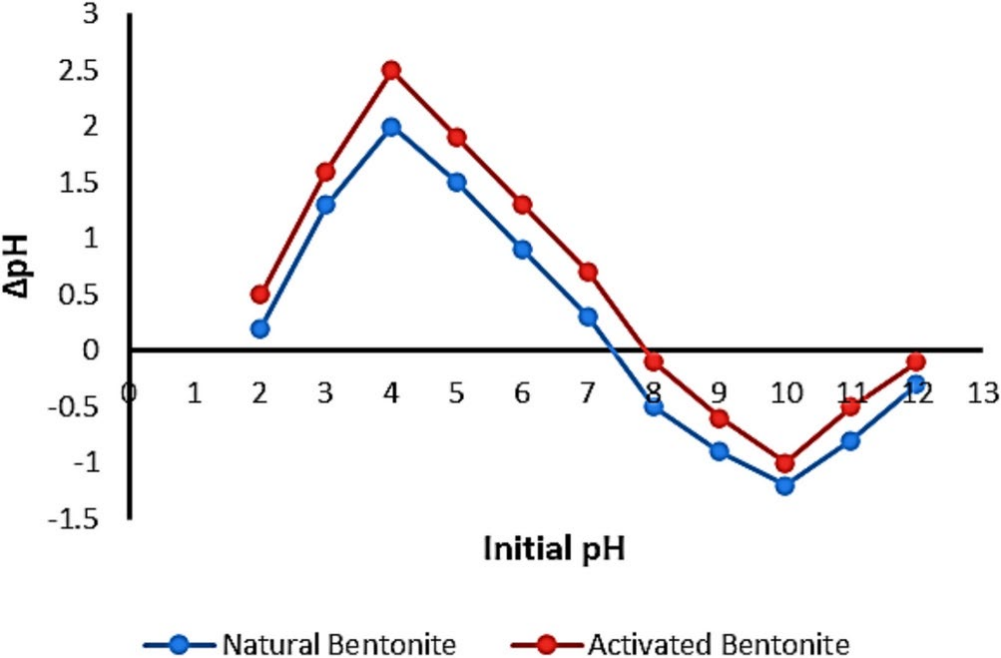
Pzc determination for natural and activated bentonite.

Figure 5 depicts the point of zero charge (pzc) assessment for both natural and activated bentonite using the pH drift method. The pzc, defined as the pH at which the surface has no net charge (ΔpH = 0), was determined to be 7.3 for natural bentonite and 7.9 for activated bentonite. This parameter is essential for understanding how bentonite’s surface charge changes with pH. When the solution pH is below the pzc, protonation of surface hydroxyl groups occurs, creating a net positive charge on the edge sites, while the basal surfaces remain negatively charged. This leads to electrostatic repulsion, reducing metal cation adsorption. In contrast, at pH levels above the pzc, deprotonation of surface hydroxyl groups results in a net negative surface charge, which strengthens as pH increases. This negative charge enhances electrostatic attraction, facilitating metal cation adsorption.

#### Scanning electron microscope (SEM)

**Fig. 6:**
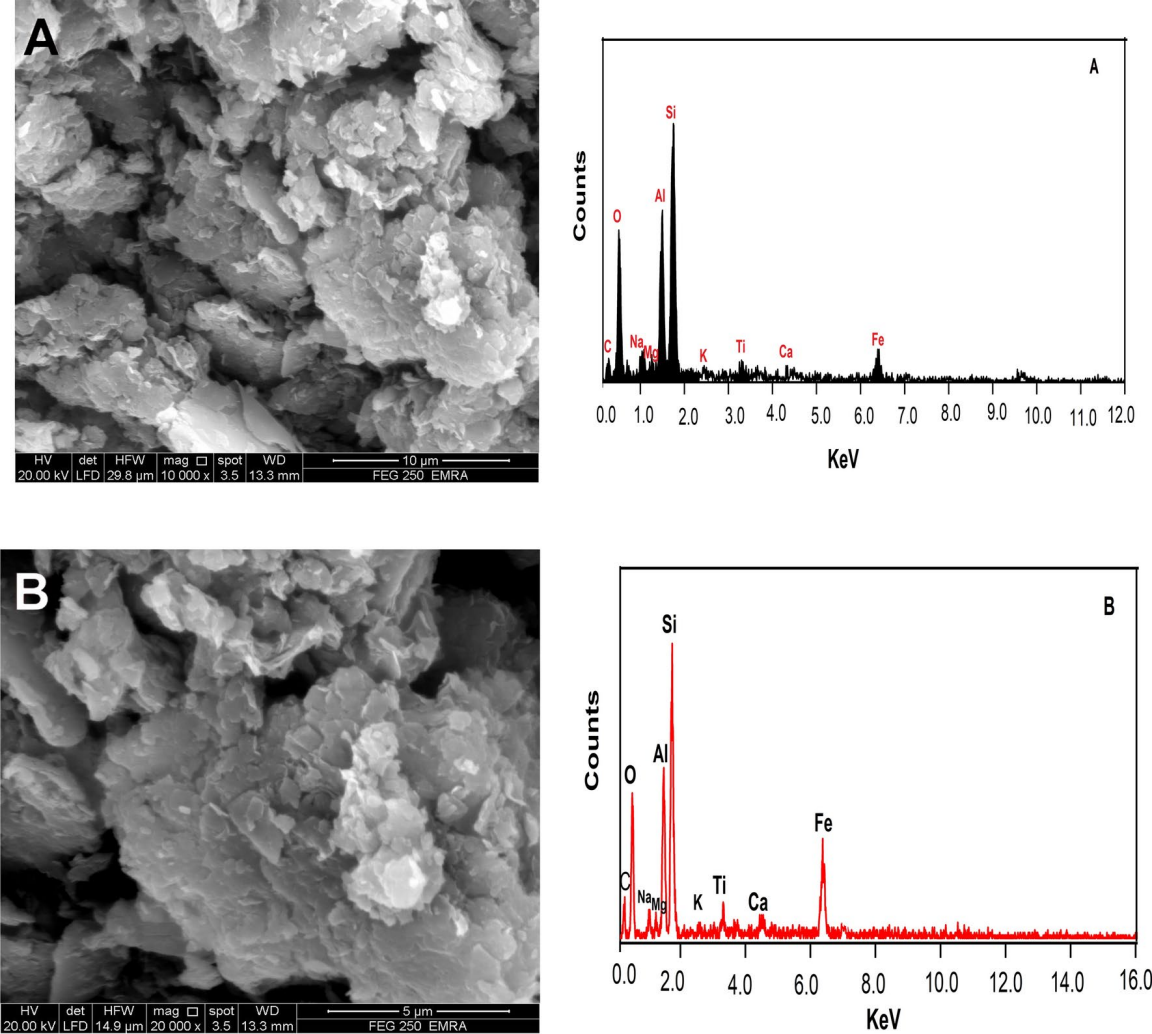
SEM & EDX images of (**A**) activated and (**B**) natural bentonite.

Figure 6 presents SEM images and EDX spectra of natural and activated bentonite, providing insight into the adsorption mechanism. Natural bentonite has a dense and less porous structure, while activation results in a more fragmented and highly porous form. The alkali activation process alters the material’s structure, expanding its surface area and increasing the number of active adsorption sites, thereby improving its capacity to trap heavy metal ions. The enhanced porosity of activated bentonite promotes better metal ion diffusion, ensuring greater interaction with adsorption sites^24^. Additionally, the rougher surface texture and increased pore volume create more opportunities for ion exchange and surface complexation, enhancing its efficiency in adsorbing Cu^2^⁺, Pb^2^⁺, and Ni^2^⁺. The EDX spectra confirm the elemental makeup of the samples, indicating significant changes in element distribution post-activation.

### Adsorption studies using natural and activated bentonite samples

#### Effect of pH

To examine the impact of pH on the adsorption of copper, lead, and nickel onto bentonite clay, a series of single-component aqueous solutions (50 mL each) containing 20 mg/L of Cu^2^⁺, Pb^2^⁺, and Ni^2^⁺ were prepared at varying pH levels ranging from 1 to 9. The pH adjustments were made using HCl and NaOH. A 0.05 g portion of both natural and activated bentonite (equivalent to 1 g/L) was introduced as the adsorbent, and the mixtures were agitated at 20 °C for 120 min in a water bath shaker. The remaining metal concentrations in the solutions were analyzed using a GBC atomic absorption SavantAA spectrometer after generating a reference calibration curve. The adsorption capacities of activated bentonite for each metal ion were determined as follows: Cu^2^⁺; 2.6 ± 0.01, 4.6 ± 0.03, 10.6 ± 0.03, 14 ± 0.03, and 19 ± 0.05 mg/g; Pb^2^⁺; 2.2 ± 0.03, 3.4 ± 0.02, 9.2 ± 0.04, 13 ± 0.04, and 18.4 ± 0.05 mg/g; and Ni^2^⁺;1.4 ± 0.008, 2.9 ± 0.02, 8.16 ± 0.03, 12.2 ± 0.05, and 15 ± 0.04 mg/g at pH levels of 1, 3, 5, 7, and 9, respectively. The results indicated that activated bentonite exhibited superior adsorption performance compared to its natural counterpart.

**Fig. 7:**
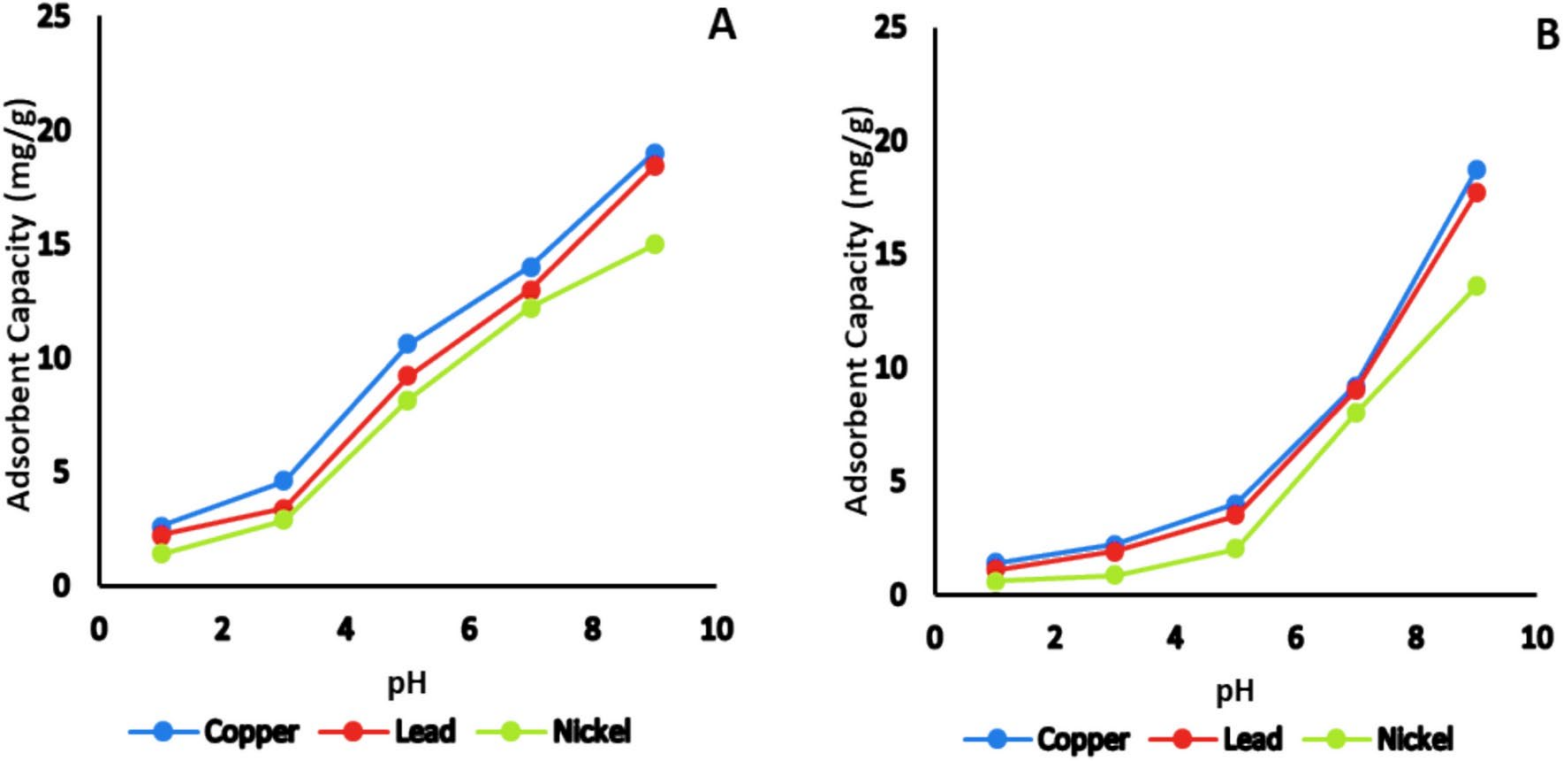
Effect of pH on the adsorption capacity of Cu^2+^, Pb^2+^ and Ni^2+^ on (**A**): activated and (**B**): natural bentonite.

Overall, adsorption capacity was observed to decrease at lower pH levels. This trend can be attributed to the competitive interaction between metal cations and hydronium (H_3_O⁺) ions for available adsorption sites on the bentonite surface. At highly acidic conditions, the abundance of H_3_O⁺ ions overshadow metal ions, leading to surface saturation with H_3_O⁺, thereby reducing metal uptake. As pH increases, H_3_O⁺ ions gradually vacate the surface, making more binding sites accessible for metal ions, ultimately enhancing their adsorption onto bentonite^25^. The sharp increase of adsorption of heavy metals at pH > 8 may be because of their hydroxide precipitation on adsorbent surface and these results agreed with results of previous study^26^. The adsorbent capacity of the examined heavy metals was determined in the following order: Cu^2+^  > Pb^2+^  > Ni^2+^, as seen in Fig. 7.

#### Effect of the adsorbent dose

**Fig. 8:**
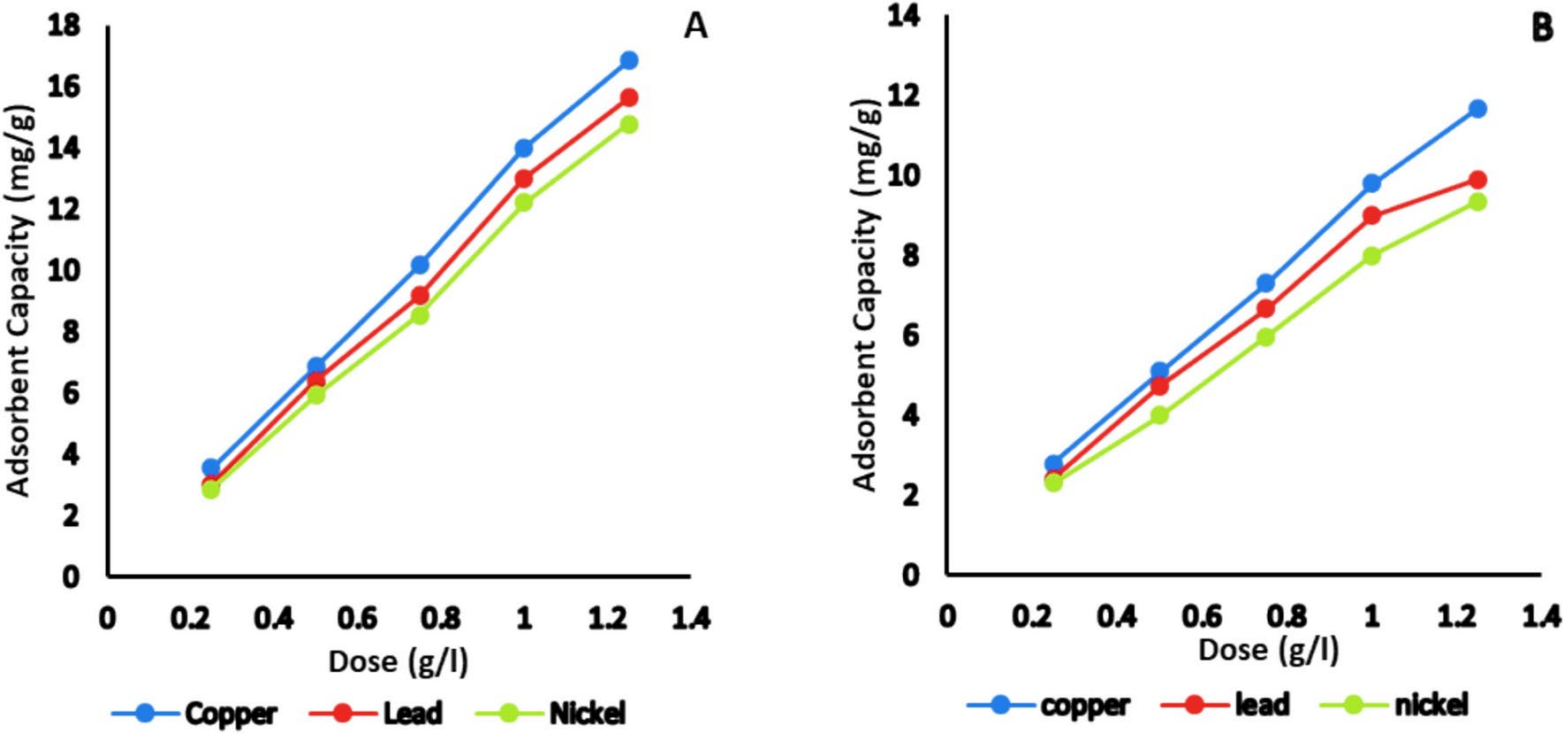
Effect of initial adsorbent dosage on the adsorption capacity of Cu^2+^, Pb^2+^and Ni^2+^ on (**A**): activated and (**B**): natural bentonite.

The effect of adsorbent dosage was studied to identify the optimal amount for heavy metal removal. The findings indicate that increasing the adsorbent dose enhances the adsorption capacity, as anticipated, owing to the increased availability of adsorption sites on the adsorbent surface. Concentrations of natural and activated bentonite were tested at 0.25, 0.5, 0.75, 1, and 1.25 g/L, using 50 mL of a 20 mg/L single solution of Cu^2+^, Pb^2+^ and Ni^2+^ at pH 7 and 20 °C for 120 min, as shown in Fig. 8. The residual solution was analyzed using GBC AA. It was found that increasing the adsorbent dosage from 0.25 to 1.25 g/L significantly improved the adsorption capacities (3.56 ± 0.04, 6.86 ± 0.03, 10.18, 14 ± 0.5 and 16.84 ± 0.05 mg/g for Cu^2+^, 3.04 ± 0.02, 6.38 ± 0.03, 9.18 ± 0.04, 13 ± 0.04 and 15.62 ± 0.05 mg/g for Pb^2+^ and 2.86 ± 0.03, 5.92 ± 0.04, 8.54 ± 0.04, 12.2 ± 0.05 and 14.76 ± 0.04 mg/g for Ni^2+^), which can be attributed to the increased availability of exchangeable sites in the bentonite clay structure^27^. The findings demonstrated that the activation of bentonite enhanced the adsorption capacity and removal efficiency.

#### Effect of contact time

**Fig. 9:**
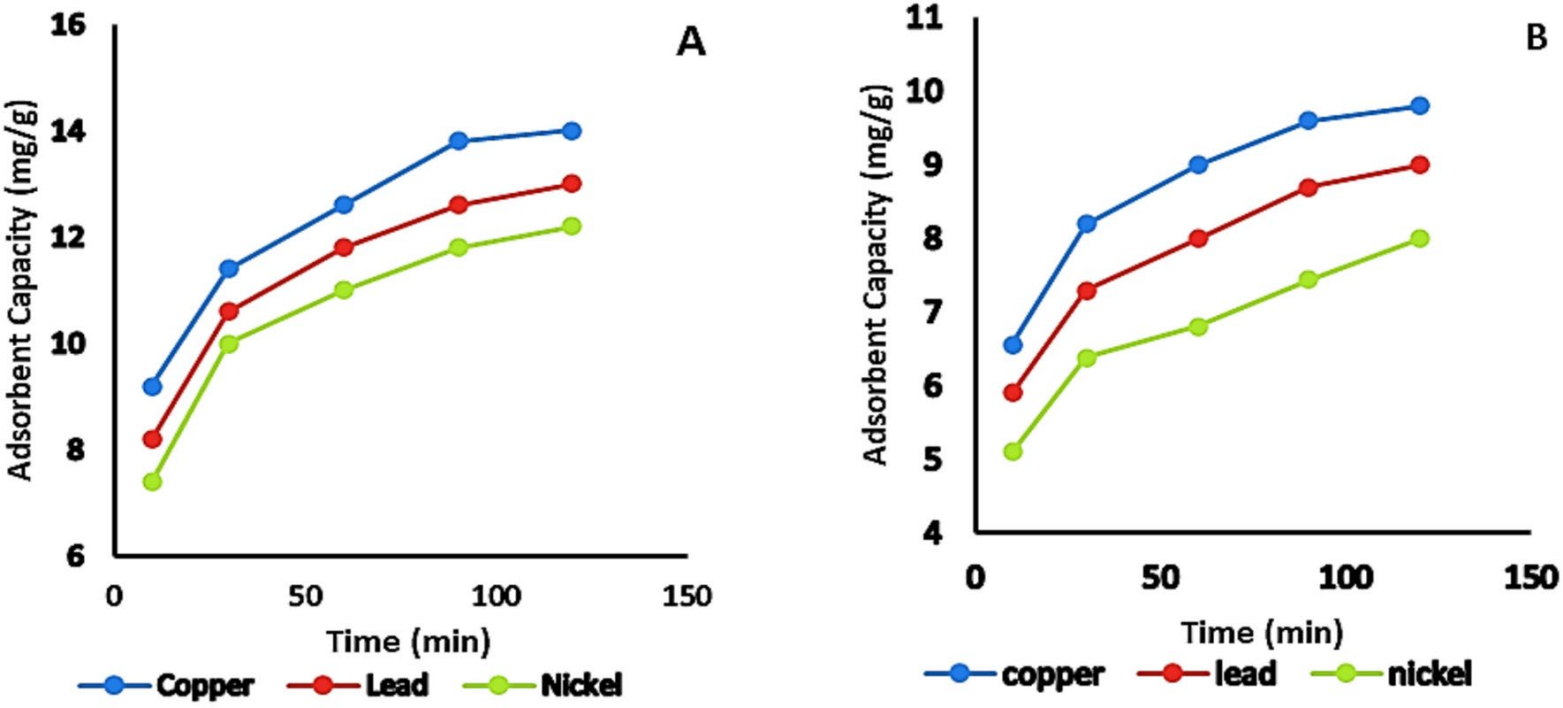
Effect of contact time on the adsorbent capacity of Cu^2+^, Pb^2+^ and Ni^2+^ on (**A**): activated and (**B**): natural bentonite.

The adsorption capacity of activated bentonite steadily increased with time, reaching equilibrium at 120 min under controlled conditions: 50 mL of a 20 mg/L single-metal solution, pH 7, a temperature of 20 °C, and an adsorbent dosage of 0.05 g (1 g/L), as illustrated in Fig. 9. The influence of contact time on Cu^2+^, Pb^2+^, and Ni^2+^ adsorption was examined at different time intervals. The results indicated a rapid adsorption rate within the first 20 min, aligning with previous studies that have reported swift metal ion uptake within 10–20 min.

At the initial phase of adsorption, a large number of available surface sites facilitate metal ion binding. However, as time progresses, occupying the remaining vacant sites becomes more challenging due to repulsive interactions between the solute molecules. The quick electrostatic attraction of heavy metal ions at neutral pH is primarily due to the negatively charged surface of the bentonite, which enhances metal ion removal from the solution^18^. At equilibrium, after 120 min at 20 °C, the adsorption capacities were 9.2 ± 004, 11.4 ± 0.03, 12.6 ± 0.04, 13.8 ± 0.02 and 14 ± 0.03 mg/g for Cu^2+^, 8.2 ± 0.03, 10.6 ± 0.02, 11.8 ± 0.04,12.6 ± 0.05 and 13 ± 0.04 mg/g for Pb^2+^, and 7.4 ± 0.01, 10 ± 0.03, 11 ± 0.04, 11.8 ± 0.04 and 12.2 ± 0.05 mg/g for Ni^2+^, respectively, with activated bentonite which showed improvement more than natural bentonite.

#### Effect of initial metal ion concentration

**Fig. 10:**
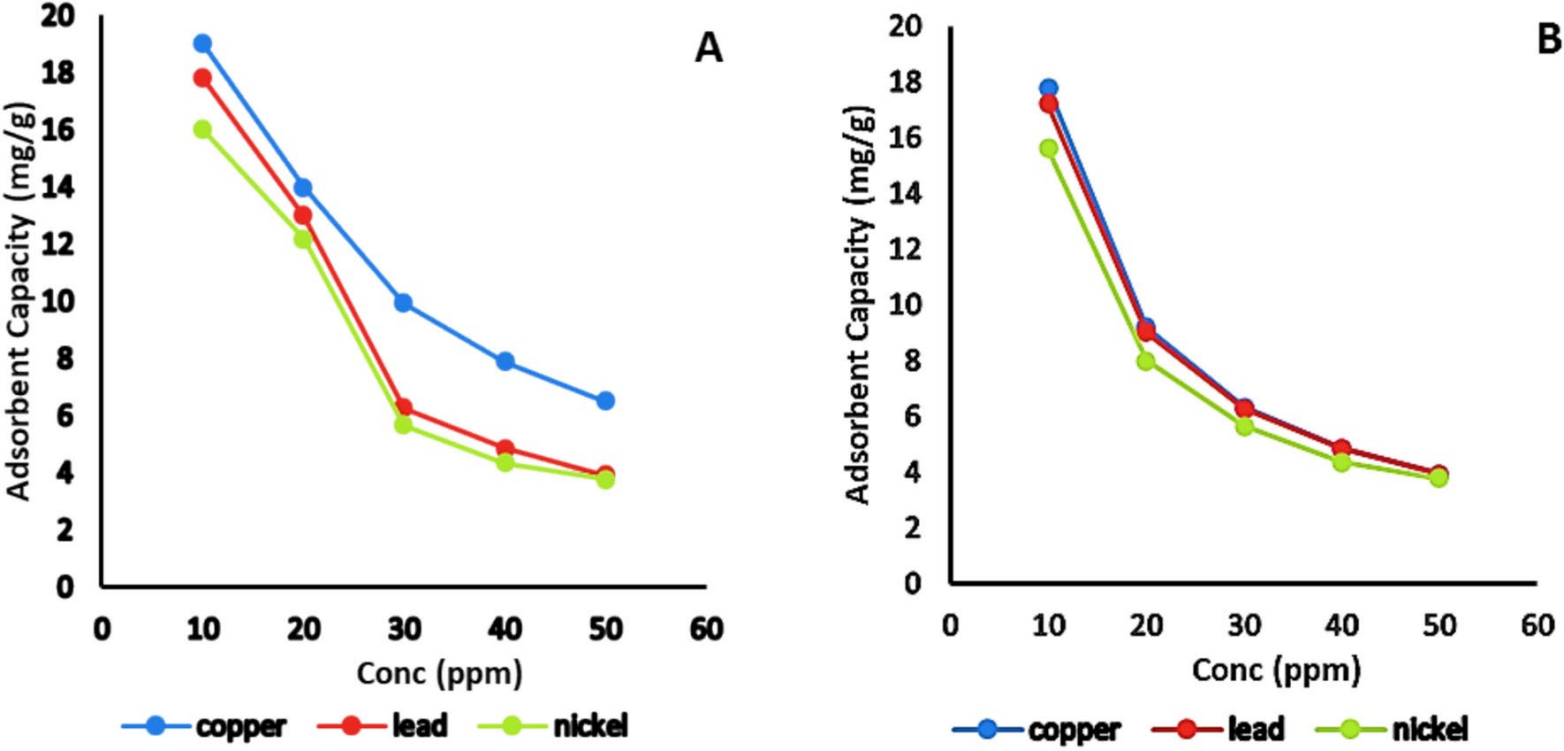
Effect of initial metals concentration on the adsorption capacity of Cu^2+^, Pb^2+^ and Ni^2+^ on (**A**): activated and (**B**): natural bentonite.

The findings indicated that the adsorption capacity (mg/g) diminished as the initial concentration increased, as seen in Fig. 10. It was observed that adsorption capacity of heavy metals on the activated and natural bentonite samples decreased by increasing the initial metal concentration (50 ml of 10, 20, 30, 40, 50 mg/L of single component solutions) and these results agreed with previous study^28^ While keeping the other variables constant (1 g/L bentonite, 20 °C, pH 7, and 120 min), this could be attributed to the insufficient number of active sites available for interaction with the adsorbate. The adsorption capacity of activated bentonite was 19 ± 0.04, 14 ± 0.03, 9.94 ± 004, 7.9 ± 0.02 and 6.52 ± 0.03 mg/g for Cu^2+^, 17.8 ± 0.04, 13 ± 0.04, 6.27 ± 0.03, 4.85 ± 0.05 and 3.92 ± 0.04 for Pb^2+^, and 16 ± 0.05, 12.2 ± 0.05, 5.67 ± 0.04, 4.35 ± 0.03 and 3.76 ± 0.04 mg/g for Ni^2+^.

#### Effect of temperature

**Fig. 11:**
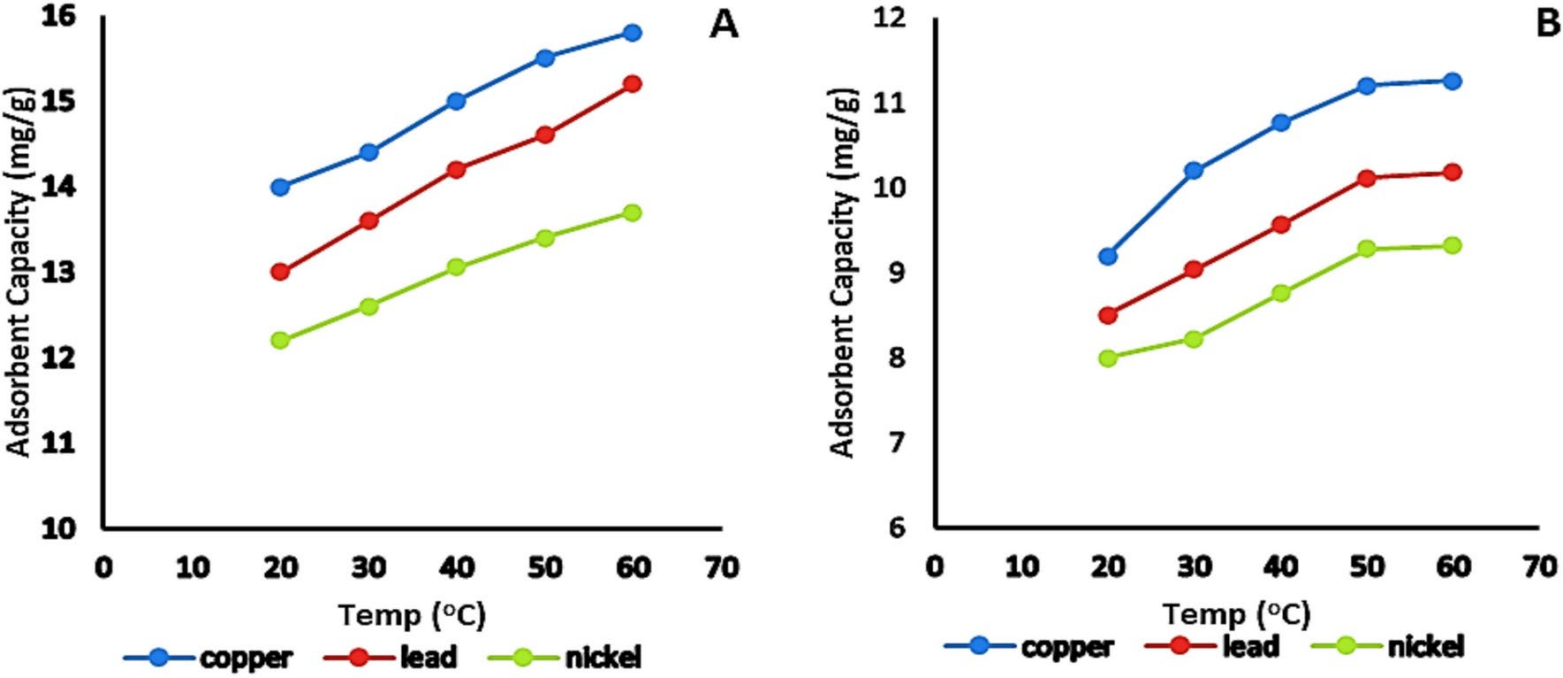
Effect of initial temperature on the adsorption capacity of Cu^2+^, Pb^2+^ and Ni^2+^ on (**A**): activated and (**B**): natural bentonite.

The adsorption efficiency of activated bentonite for heavy metals improved with increasing temperature, likely due to enhanced molecular interactions and accelerated reaction kinetics at the adsorbent-adsorbate interface. Higher temperatures may also induce structural expansion in the bentonite layers, facilitating metal ion diffusion^29^. This effect was examined by adjusting the temperature to 20, 30, 40, 50, and 60 °C while maintaining constant parameters: 50 mL solution containing 20 mg/L of heavy metal, pH 7, an adsorbent dose of 1 g/L, and a contact period of 120 min. The adsorption capacities recorded for Cu^2^⁺ were 14 ± 0.03, 14.4 ± 0.06, 15 ± 0.05, 15.5 ± 0.06, and 15.8 ± 0.04 mg/g; for Pb^2^⁺, 13 ± 0.04, 13.6 ± 0.06, 14.2 ± 0.05, 14.6 ± 0.03 and 15.2 ± 0.05 mg/g; and for Ni^2^⁺, 12.2 ± 0.05, 12.6 ± 0.04, 13.06 ± 0.05, 13.4 ± 0.06 and 13.7 ± 0.05 mg/g, as illustrated in Fig. 11.

### Adsorption kinetics

To better understand the mechanism of heavy metal adsorption onto activated bentonite, the subsequent kinetic models were employed to interpret the experimental results.

#### Pseudo first order model

**Fig. 12:**
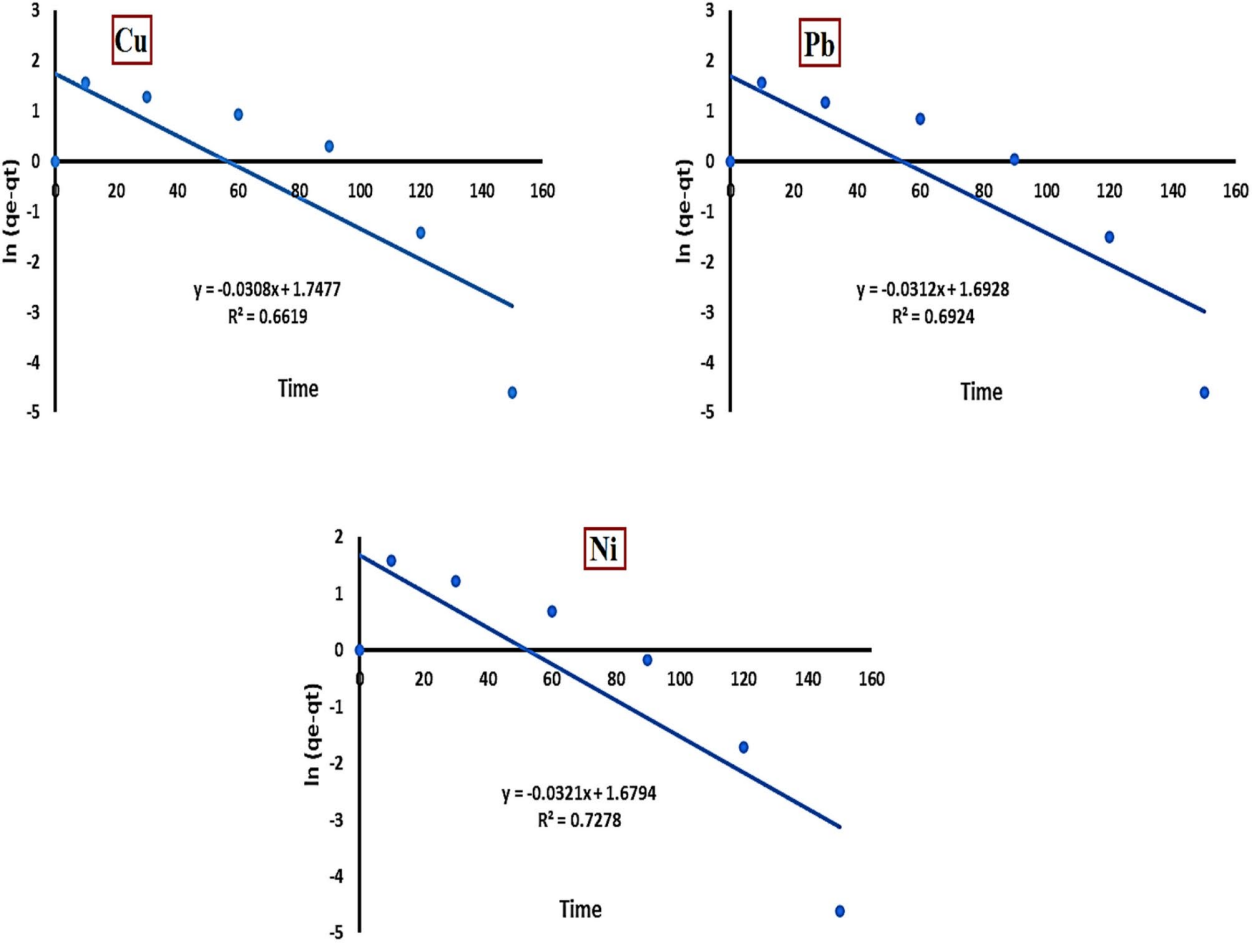
Pseudo first order plot for Cu^2+^, Pb^2+^, and Ni^2+^ adsorption onto activated bentonite.

This kinetic model can be expressed as follows^30^:

4 $$\text{ln}\left({q}_{e}-{q}_{t}\right)= \text{ln}{q}_{e}-{K}_{1} t$$

where q_e_ and q_t_ denote the quantity of metal ion adsorbed on the adsorbent (mg/g) at equilibrium and at time t, respectively, K1 signifies the rate constant for first-order adsorption (min^-1^). By plotting ln (q_e_–q_t_) against time (t), a linear relationship is obtained, from which the first-order rate constant (K1) and equilibrium adsorption capacity (q_e_) are determined from the slope and intercept, respectively. As shown in Fig. 12, the R^2^ values for Cu^2^⁺, Pb^2+^, and Ni^2+^ are 0.6619, 0.6924, and 0.7278, respectively.

#### Pseudo second order model

The adsorption data were analyzed using the pseudo-second-order model, represented by the following equation^30^:

5 $$\frac{t}{{q}_{t}}=\frac{t}{{q}_{e}}+\frac{1}{{k}_{2}{{q}_{e}}^{2}}$$

**Fig. 13:**
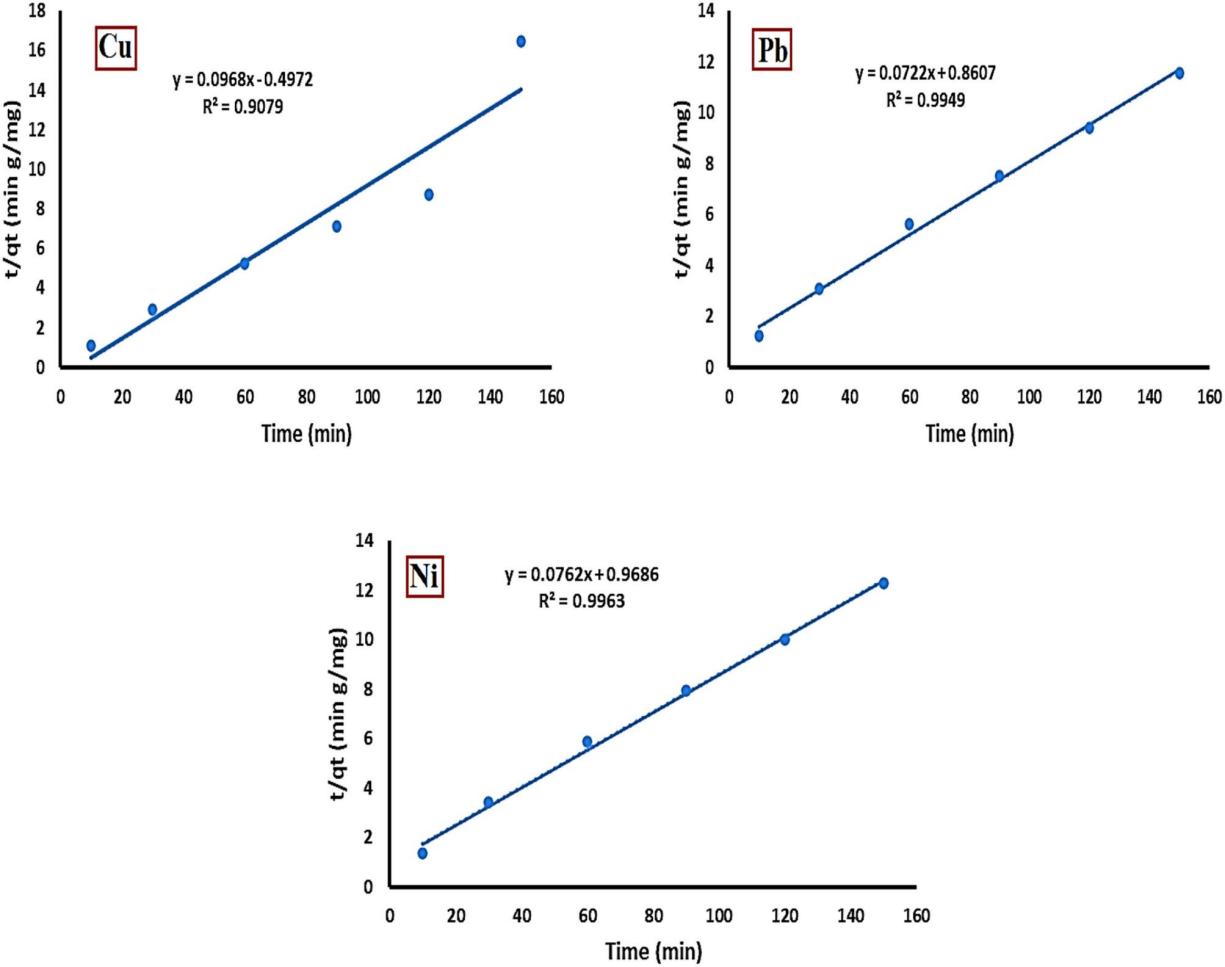
Pseudo second order plot for Cu^2+^, Pb^2+^, and Ni^2+^ adsorption onto activated bentonite.

Figure 13 demonstrates that the correlation coefficient (R^2^) values were 0.9079 for Cu^2+^, 0.9949 for Pb^2+^, and 0.9963 for Ni^2+^. The results indicate that the adsorption of Cu^2+^, Pb^2+^, and Ni^2+^ ions onto the activated bentonite surface conforms to the pseudo-second-order model. This implies that the adsorption process is primarily controlled by chemical sorption, suggesting the involvement of valence forces, ion exchange, or covalent bond formation^31^.

#### Adsorption isotherm models

The adsorption isotherm illustrates the equilibrium relationship between the concentration of the adsorbate in the liquid phase and its concentration on the adsorbent surface under specific conditions. Langmuir and Freundlich isotherm models were used to establish the relationship between the amounts of Cu^2+^, Pb^2+^, and Ni^2+^ ions adsorbed by activated bentonite clay and their equilibrium concentrations in the aqueous solution.

#### Langmuir isotherm model

The linear representation of the Langmuir isotherm model, which characterizes monolayer adsorption on a surface with a finite number of uniform binding sites, is articulated by the subsequent equation^32^:

6 $$\frac{{c}_{e}}{{q}_{e}}= \frac{1}{{k}_{L}{q}_{max}}+ \frac{{c}_{e}}{{q}_{max}}$$

**Fig. 14:**
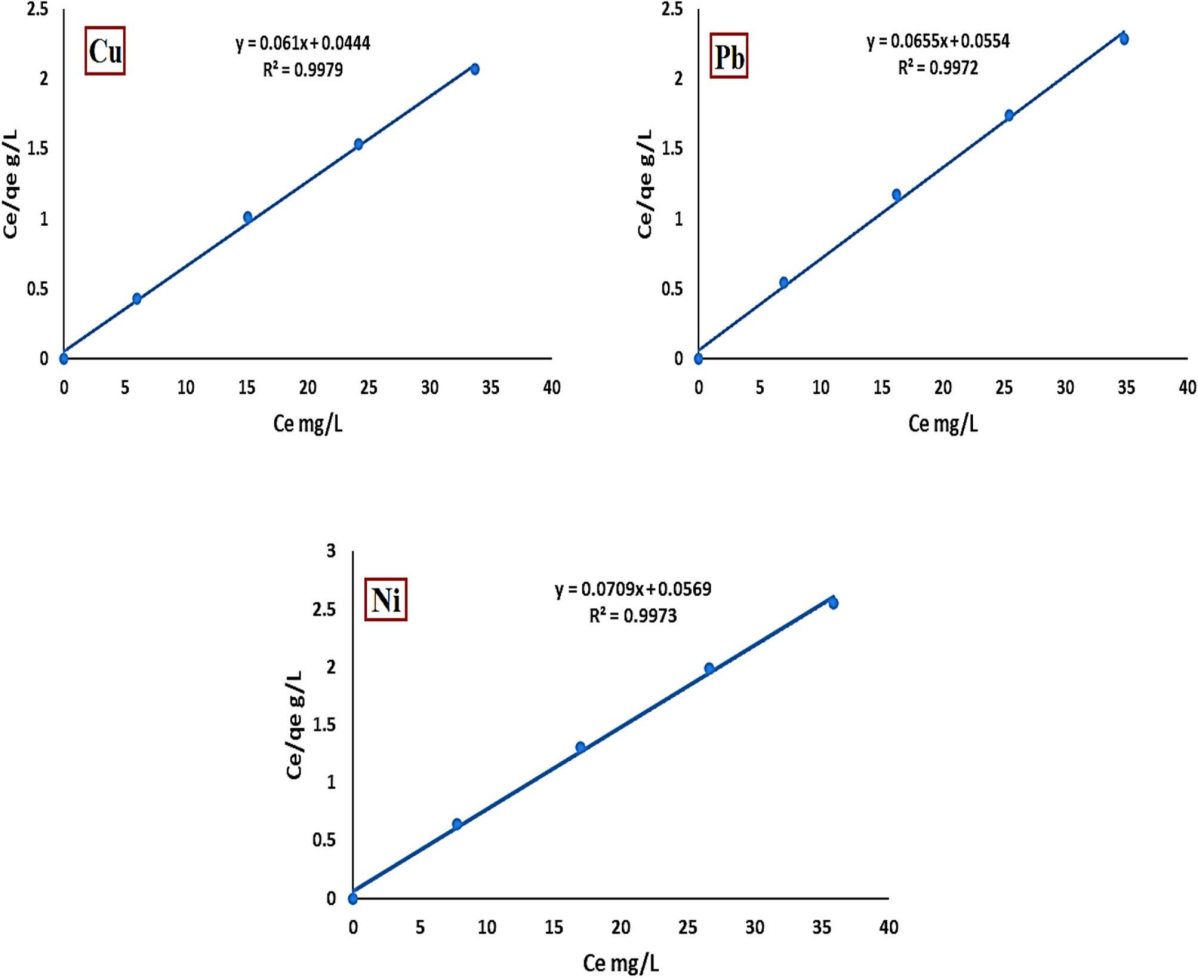
Langmuir plot of Cu^2+^, Pb^2+^and Ni^2+^ onto activated bentonite.

In this context, qmax signifies the maximum monolayer adsorption capacity (mg/g), Ce indicates the equilibrium concentration of metal ions in solution (mg/L), q_e_ refers to the amount of metal ions adsorbed per gram of activated bentonite at equilibrium (mg/g), and KL represents the Langmuir constant associated with the affinity of the binding sites (L/mg). The Langmuir isotherm assumes the formation of a single monolayer during the reaction, with fixed positions for the adsorbate, no interaction between the adsorbate and adsorbent, and no overlap between adsorbate molecules on the surface^33^. The correlation coefficients for the Langmuir isotherm were 0.9979 for copper, 0.9972 for lead, and 0.9973 for nickel, as shown in Fig. 14. This signifies that the Langmuir isotherm effectively characterizes Cu^2+^, Pb^2+^, and Ni^2+^ adsorption onto bentonite clay, implying that the adsorption process transpires as anticipated. The calculated maximum monolayer capacities (q_max_) were 14 ± 0.03 mg/g for Cu^2+^, 13 ± 0.04 mg/g for Pb^2+^, and 12.2 ± 0.05 mg/g for Ni^2^⁺.

The Langmuir isotherm’s essential properties can be represented by R_L_, a dimensionless constant referred to as the separation factor or equilibrium parameter. The value of R_L_ is determined using the following equation^34^:

7 $${R}_{L}= \frac{1}{1+{K}_{L}{c}_{0}}$$

**Fig. 15:**
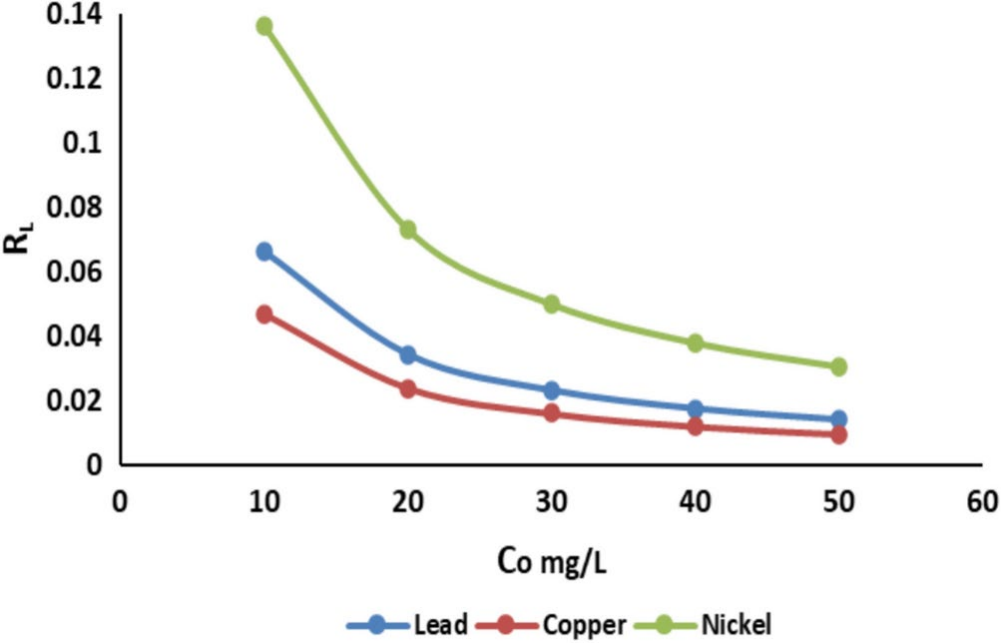
The Separation factor R_L_ versus initial concentration for Cu^2+^, Pb^2+^, and Ni^2+^ adsorption onto activated bentonite.

Co indicates the initial concentration of metal ions (mg/L), whereas K_L_ signifies the Langmuir adsorption equilibrium constant (L/mg). The R_L_ parameter serves as a more reliable indicator of the adsorption process. It specifies the arrangement of the isotherm as follows: unfavorable (R_L_ > 1), linear (R_L_ = 1), favorable (0 < R_L_ < 1), or irreversible (R_L_ = 0)^35^. The computed R_L_ values for Cu^2+^, Pb^2+^, and Ni^2+^ in this investigation were determined to be within the interval of 0 < R_L_ < 1. The R_L_ varied from 0.0097 to 0.0466 for Cu^2+^; while it ranged from 0.014 to 0.066 for Pb^2+^, and it spanned from 0.0306 to 0.1361 for Ni^2+^, as seen in Fig. 15.

#### Freundlich isotherm model

The following equation expresses the linear form of the Freundlich model^36^:

8 $$log \, q_{e} \, = \,log \, K_{F} \, + \,1/n \, log \, C_{e}$$

**Fig. 16:**
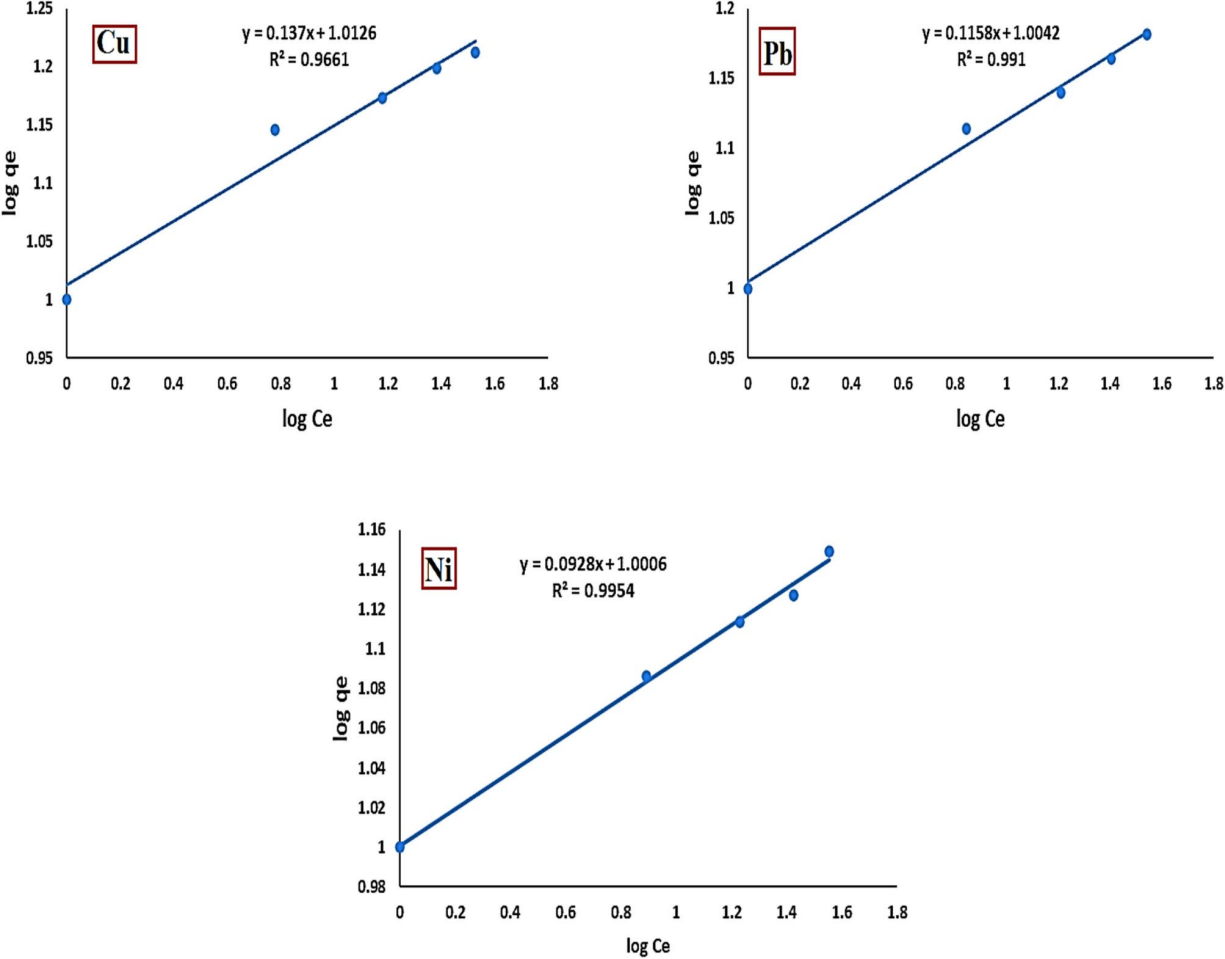
Freundlich plot of Cu^2+^, Pb^2+^and Ni^2+^ onto activated bentonite.

As presented in Fig. 16, the correlation coefficients (R^2^) for Cu^2+^, Pb^2+^, and Ni^2+^ were 0.9661, 0.9910, and 0.9954, respectively. These values suggest that the Freundlich isotherm model provides a less accurate fit for the adsorption data. Therefore, the Langmuir isotherm provided a better fit for the adsorption of Cu^2+^, Pb^2+^, and Ni^2^⁺.

### Thermodynamics studies

**Fig. 17:**
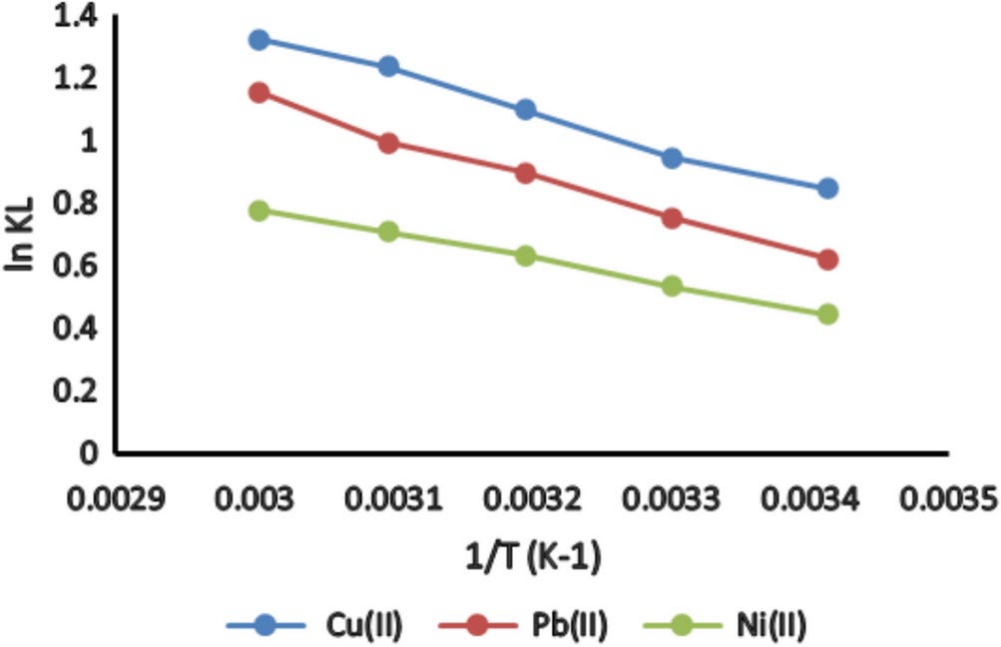
Thermodynamic behaviors of adsorption of Cu^2+^, Pb^2+^ and Ni^2+^ onto activated bentonite.

The thermodynamic parameters ΔH° and ΔS° were determined from the slope and intercept of the Van’t Hoff plot of ln K_L_ versus 1/T (Fig. 17). The negative values of Gibbs free energy change (ΔG°) for Cu^2+^, Pb^2+^, and Ni^2+^ adsorption confirm that the process is spontaneous and thermodynamically favorable. These values also indicate the presence of electrostatic interactions between the metal ions and the activated bentonite surface. Furthermore, the magnitude of ΔG° reflects the strength of metal ion binding, where more negative values correspond to stronger interactions. This adsorption behavior is influenced by the electrical, chemical, and structural properties of activated bentonite. The enthalpy change (ΔH°) provides insights into the energy dynamics of the adsorption process^37^. The positive ΔH° values for Cu^2+^, Pb^2+^, and Ni^2+^ as illustrated in Table 3 confirm that the process is endothermic, meaning that adsorption efficiency improves with increasing temperature. Similar studies have been previously observed^38^. In general, ΔH° values below 20 kJ/mol suggest a physical adsorption mechanism, while values exceeding this range indicate additional processes such as ion exchange, which involves weak chemical interactions. The entropy change (ΔS°) provides further insight into adsorption behavior, particularly in terms of molecular arrangement. A negative ΔS° suggests that metal ions assume a more ordered configuration on the bentonite surface, leading to reduced system randomness. Conversely, a positive ΔS° implies structural modifications in both the adsorbent and adsorbate during the adsorption process. Overall, the thermodynamic analysis confirms that heavy metal adsorption onto activated bentonite is spontaneous, endothermic, and influenced by electrostatic interactions and ion exchange.

**Table 3:** Thermodynamic parameters of Cu^2+^, Pb^2+^and Ni^2+^ adsorption on activated bentonite Clay.

T (K)	Metal ion	ΔH° (KJ/mol)	ΔS° (KJ/K.mol)	ΔG° (KJ/mol)
293	Cu^2+^	10.129045	0.041487	− 2.06054
303				− 2.37783
313				− 2.8589
323				− 3.31776
333				− 3.66674
293	Pb^2+^	10.58183	0.041222	− 1.51173
303				− 1.89886
313				− 2.33187
323				− 2.6673
333				− 3.19418
293	Ni^2+^	6.826873	0.027001	− 1.08326
303				− 1.34117
313				− 1.64551
323				− 1.90137
333				− 2.15125

## Mechanism of Pb, Cu, and Ni adsorption on activated and natural bentonite

Several key processes influence the adsorption of Cu^2+^, Pb^2+^, and Ni^2+^ onto natural bentonite (NB) and activated bentonite (AB), including ion exchange, surface complexation, and electrostatic interactions. Alkali activation significantly enhances the cation exchange capacity (CEC) of AB, allowing for more efficient metal ion exchange with sodium ions on the bentonite surface^39^. Surface complexation occurs as metal ions interact with functional groups such as hydroxyl (–OH) and silanol (Si–OH), facilitating stronger adsorption. Electrostatic attraction also plays a vital role, as the negatively charged bentonite surface attracts positively charged metal ions, particularly at neutral to slightly alkaline pH conditions. The adsorption process follows the Langmuir isotherm model, indicating monolayer adsorption on a finite number of binding sites. The adsorption kinetics follow the pseudo-second-order model, indicating that chemisorption is the dominant mechanism involving electron sharing or transfer between metal ions and bentonite functional groups. Thermodynamic analysis confirms that the process is spontaneous and endothermic, with higher temperatures enhancing metal ion uptake. These combined mechanisms contribute to the enhanced heavy metal removal efficiency of activated bentonite compared to its natural form. Table 4 presents a comparative analysis of Cu^2+^, Pb^2+^, and Ni^2+^ adsorption capacities across various adsorbents, highlighting the superior performance of activated bentonite in heavy metal removal.

**Table 4:** Adsorption capacities of Cu^2+^, Pb^2+^and Ni^2+^ on different adsorbents.

Adsorbent	Pb^2+^ Adsorption (mg/g)	Ni^2+^ Adsorption (mg/g)	Cu^2+^ Adsorption (mg/g)	Refs.
Activated Bentonite (AB)	13 ± 0.04	12.2 ± 0.05	14 ± 0.03	This Study
Natural Bentonite (NB)	9 ± 0.03	8 ± 0.02	9.2 ± 0.04	This Study
Zeolite	–	–	5.1	^40^
Luffa Actangula Carbon	–	–	12.47	^41^
Activated rubber wood sawdust	–	–	5.73	^42^
Chai teabags	–	–	16.28	^43^
Peat	–	–	7.39	^44^
Na bentonite	–	13	–	^26^
Kaolinite	–	1.67	–	^45^
Activated carbon	–	14.65	–	^46^
red mud	–	13.69	–	^47^
Activated carbon	–	5.4	–	^48^
Carbon aerogel	–	2.8	–	^49^
Beysehir lignite	–	12	–	^48^
Acid modified montmorillonite	–	7.31	–	^50^
Na-montmorillonite	–	10.04	–	^50^
Mg-oxide Immobilized Sand	2.843	–	–	^51^
Sawdust	3.19	–	–	^52^
Turkish low rank coal	13.58	–	–	^53^
Natural kaolinite	7.75	–	–	^54^
Acid-modified monmorillinite	1.62	–	–	^55^
Natural kaolin clay	2.35	–	–	^56^

## Present and future prospects

While this study focuses on synthetic wastewater, its findings offer valuable insights for advancing water treatment strategies in Egypt. The enhanced adsorption capacity of activated bentonite highlights its potential for use in industrial wastewater treatment, where it could aid in the removal of heavy metals before discharge into natural water bodies. Additionally, bentonite-based filtration systems could serve as an effective supplementary step in municipal water purification, especially in areas with high contamination risks.

In agriculture, heavy metals in irrigation water threaten soil integrity and crop safety. The application of bentonite-based adsorbents to treat irrigation runoff could help reduce soil contamination and support more sustainable farming practices. Although this research was conducted in controlled laboratory conditions, the results provide a foundation for scaling up the process through pilot studies to assess its effectiveness in real wastewater treatment applications. Many rural areas in Egypt lack centralized wastewater treatment facilities, making activated bentonite a cost-effective and locally accessible solution for decentralized water purification. Furthermore, it can be integrated with existing treatment methods such as coagulation, flocculation, or membrane filtration to enhance contaminant removal efficiency. Future research should focus on optimizing its application in real-world conditions and evaluating its long-term viability as an eco-friendly water treatment solution.

## Conclusion

This study is concerned with the activation of natural bentonite clay and the use of this activated bentonite clay in the treatment of aqueous solution from heavy metals (Cu^2+^_,_ Pb^2+^ and Ni^2+^). Optimum results for heavy metals removal were pH 7, 1 g/L adsorbent dose, 120 min contact time, 20 mg/L initial metals concentration and 20 °C temperature for lead, copper and nickel. The maximum adsorption capacities of the activated bentonite were determined as 14 ± 0.03 mg/g for Cu^2+^, 13 ± 0.04 mg/g for Pb^2+^, and 12.2 ± 0.05 mg/g for Ni^2+^, exceeding those of the natural bentonite, which recorded capacities of 9.2 ± 0.04 mg/g, 9 ± 0.03 mg/g, and 8 ± 0.02 mg/g, respectively. The adsorption equilibrium follows the Langmuir model, with correlation coefficients (R^2^) of 0.9979 for Cu^2+^, 0.9972 for Pb^2+^, and 0.9973 for Ni^2+^. Additionally, the pseudo-second-order model well describes the adsorption kinetics of copper, lead, and nickel onto activated bentonite, yielding R^2^ values of 0.9079 for Cu^2+^, 0.9949 for Pb^2+^, and 0.9963 for Ni^2+^.The advantages of AB include cost-effective activation using Na_2_CO_3_ instead of strong acids, high adsorption capacity that competes with or exceeds some nano-clays and untreated bentonites, and an environmentally friendly process that avoids hazardous waste generation. These factors make AB a promising material for large-scale wastewater treatment applications. Further research should focus on evaluating its efficiency in real wastewater environments to validate its practical applicability and scalability.

## Data Availability

The datasets used and/or analyzed during the current study are available from the corresponding author on reasonable request.
